# Horizontal Platelet-Rich Fibrin in Vestibuloplasty: A Case Report

**DOI:** 10.7759/cureus.65862

**Published:** 2024-07-31

**Authors:** Silvia Dal Pont, Aldo Zupi

**Affiliations:** 1 Regenerative Medicine, "Dal Pont" Dental Clinic, Belluno, ITA; 2 Oral & Maxillofacial Surgery, "Centro di Medicina" Health Network, Padova, ITA

**Keywords:** platelet concentrates, oral medicine and periodontology, vestibuloplasty, horizontal platelet-rich fibrin, platelet-rich fibrin

## Abstract

Vestibuloplasty (VP) is a surgical technique that allows the deepening of the vestibule of the mouth. The gold standard, especially if an increase in keratinized tissue (KT) is required, is represented by the free gingival graft (FGG). The need for a donor site, however, is a source of discomfort and possible complications. To overcome these aspects, numerous techniques and materials have been used. Horizontal platelet-rich fibrin (H-PRF) has been very successful in recent years in various oral surgery procedures due to its ability to promote tissue healing and regeneration. The reported case presents a new technique of VP using H-PRF, which allows avoiding the second surgical site. A 25-year-old patient with post-surgical reduction of vestibule depth and poor KT was treated with VP. The patient refused an FGG procedure. Therefore, VP was performed using an H-PRF membrane as a graft material to lengthen the vestibule and promote KT regeneration. After nine weeks, an increase in vestibule depth and KT width was evident. The use of H-PRF in VP has allowed predictable surgery without significant complications. It therefore represents an alternative to the traditional FGG to be seriously taken into consideration.

## Introduction

Vestibuloplasty (VP) is a surgical technique that allows the deepening of the vestibule of the mouth [1-3]. There are numerous procedures described, but the gold standard is represented by the free gingival graft (FGG) that, necessarily, requires a second surgical site with a consequent increase in morbidity and the risk of complications [1,4]. To overcome these limitations, the use of other biomaterials has been proposed, which, while allowing greater comfort for the patient, has determined an increase in costs without achieving the clinical results of FGG [4,5].

Platelet concentrates, and in particular platelet-rich fibrin (PRF), are already used in numerous regenerative procedures (both intraoral and extraoral), including periodontology, and maintain the advantages of biomaterials (greater comfort and less morbidity) without the disadvantage of cost; therefore, they could be considered a viable alternative to FGG [6-10].

We report a case that presented a reduced vestibular depth as sequelae of post-traumatic reconstructive surgery. The VP was required to deepen the oral vestibule and improve oral health and aesthetics. The VP technique was performed with platelet concentrates using a new and not frequently described methodology (horizontal PRF or H-PRF) to make the preparation more stable over time.

## Case presentation

**Figure 1:**
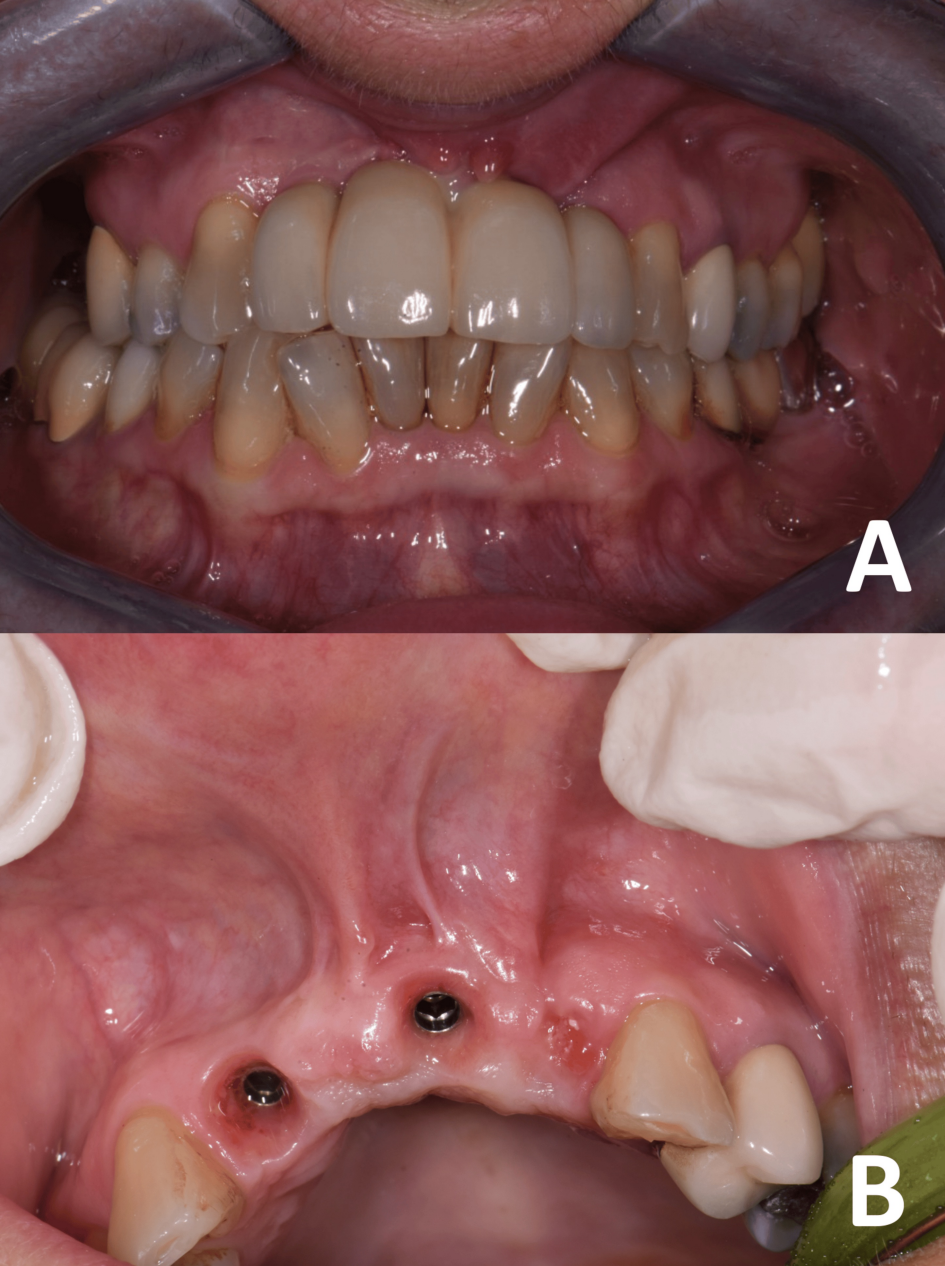
Clinical findings at presentation. The current shape of the tissues was secondary to bone regenerative surgery. (A) Note the unsatisfactory aesthetic appearance of the incisive region. (B) The vestibule appears reduced in depth with post-surgical frenulum and reduction of KT.

A 25-year-old patient reported to our observation for the evaluation of the outcomes of regenerative surgery in the second sextant. The patient reported frequent inflammatory episodes with pain, difficulty with home hygiene maneuvers, and aesthetic dissatisfaction. No relevant history was reported. On clinical examination, the reduction in length of the vestibule was evident with poor keratinized tissue (KT) and a chronic state of inflammation (Figure 1).

A VP with FGG was proposed. The presence of a second surgical site (the donor area), however, discouraged the patient. Therapeutic alternatives using biomaterials or PRF were illustrated. The patient chose the option with PRF.

**Figure 2:**
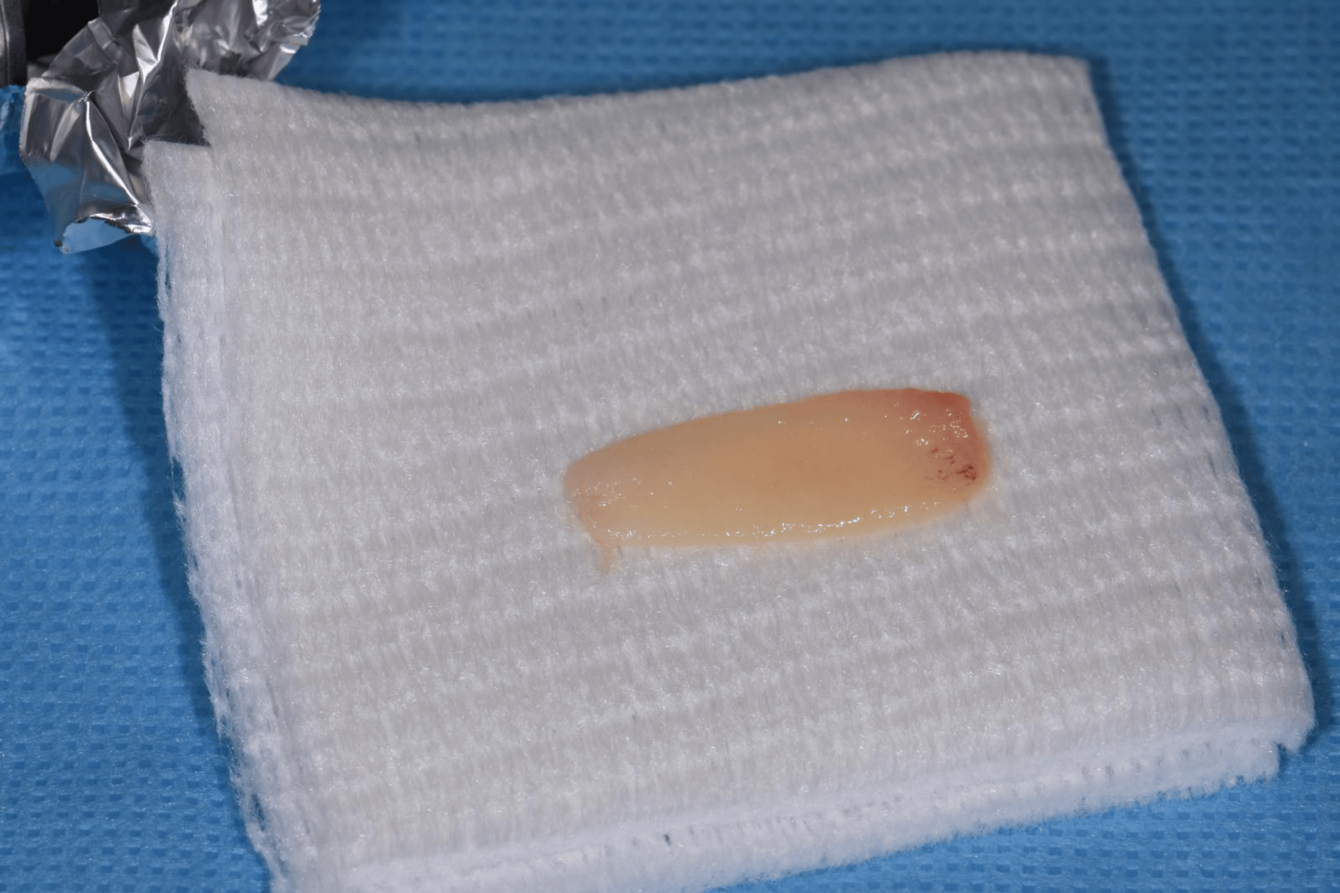
Vestibuloplasty with the H-PRF membrane. The membrane was made using H-PRF.

Before surgery, 36 mL of blood was collected and divided into four tubes without anticoagulants or other additives. The tubes were centrifuged at 700 rcf for eight minutes (Zhejiang Gongdong Medical Technology Co., Taizhou, China). The top 2 mL layer of PRF obtained through horizontal centrifugation was collected and heated at 75 °C for 10 min before mixing with the bottom 2 mL layer of PRF to obtain the membranes (Figure 2).

**Figure 3:**
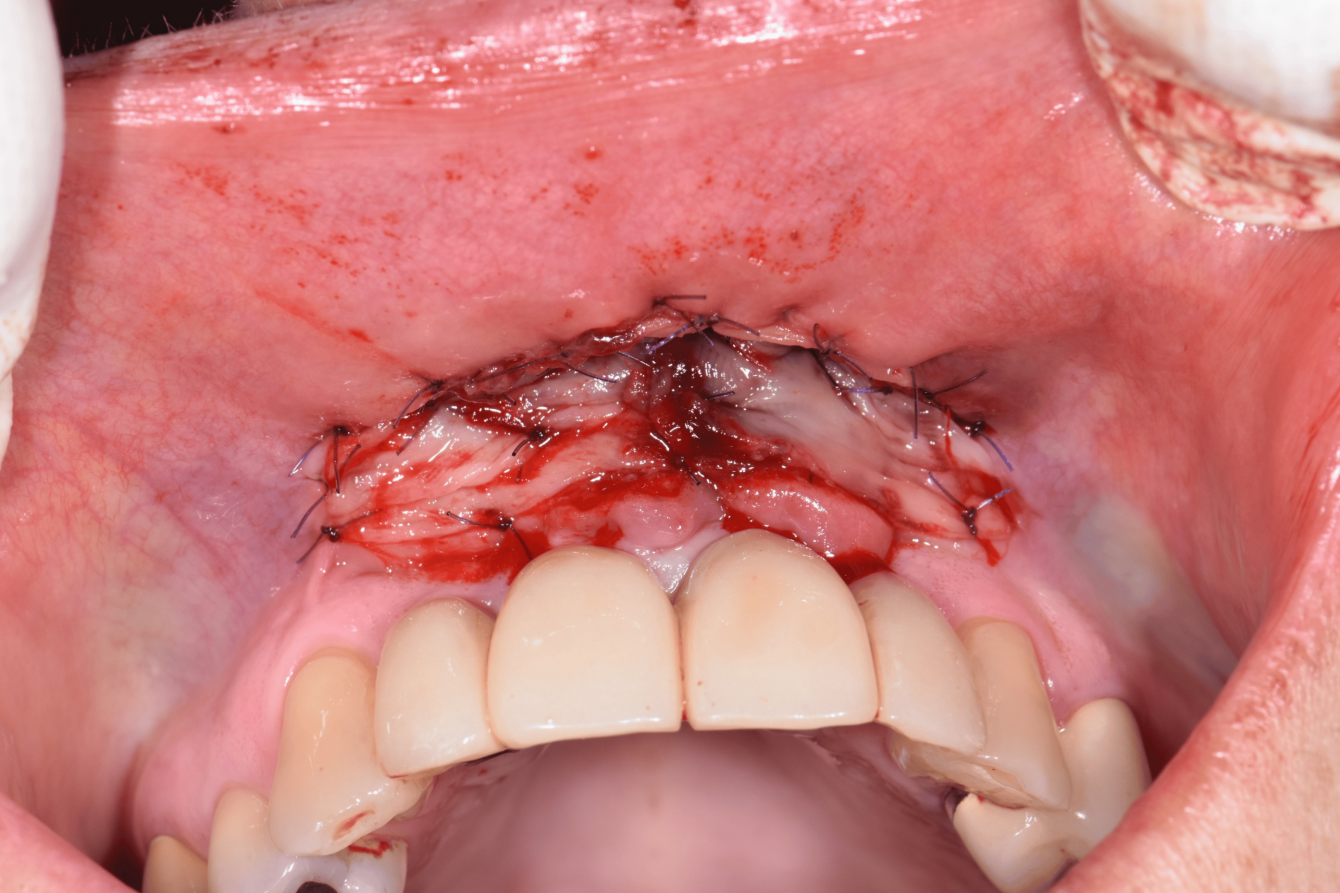
Vestibuloplasty with the H-PRF membrane. A partial thickness flap was designed and moved apically. The recipient bed was covered with the H-PRF membrane.

The surgery was performed under local anesthesia using Mepivacaine cloridrate 2% with adrenaline 1:100,000 (Carbocaina, Aspen Pharma Trading Ltd., Dublin, Ireland). A horizontal incision was made from one canine ridge to the other approximately 1-2 mm coronal to the mucogingival junction, resulting in a partial thickness flap without any releasing incision. The flap was then moved apically and sutured to the underlying periosteum with absorbable stitches (Vicryl 5.0, Ethicon; Johnson & Johnson, New Brunswick, NJ), thus creating the recipient bed for the graft. The obtained H-PRF membranes were sutured directly to the underlying periosteum (Figure 3).

Ibuprofen 600 mg per eight hours for two days, and chlorhexidine 0.20% three times per day for 15 days were prescribed. The patient was suggested a liquid and cold diet for three days, and it was recommended not to brush the area for 15 days.

**Figure 4:**
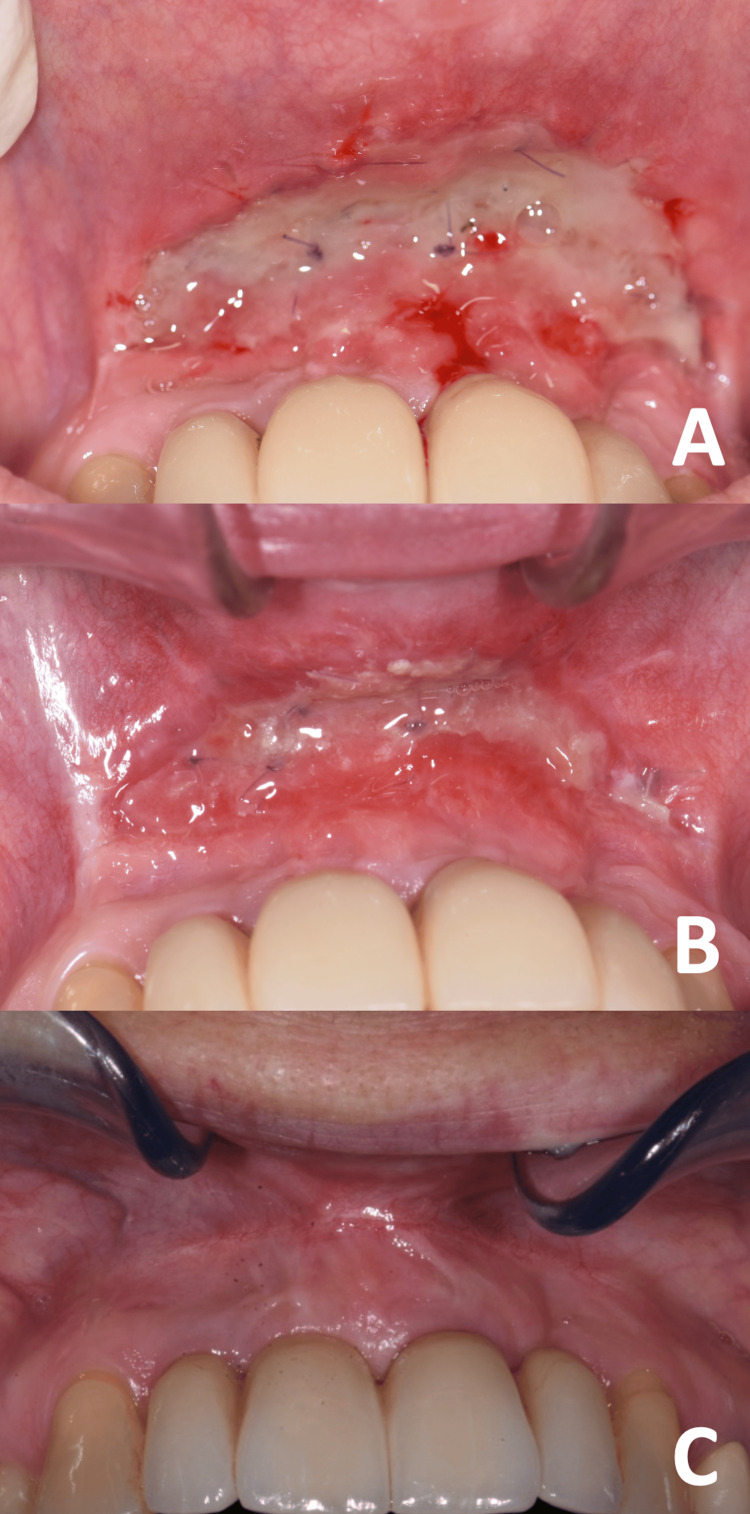
Clinical findings during follow-up. Clinical findings at (A) two weeks, (B) three weeks, and (C) six weeks. At the last follow-up, we noticed a good appearance, a deepening of the vestibule, and an increase in KT.

After two weeks, the sutures were partially removed. At this time, partial re-epithelialization of the area was observed (Figure 4A). After three weeks, the re-epithelialization was almost complete (Figure 4B). After three months, an increase in the width of KT was observed (Figure 4C). The patient reported no discomfort or recurrence of inflammatory episodes. The aesthetic appearance was judged satisfying.

## Discussion

The objective of a VP is, essentially, the increase in depth of the vestibule of the mouth and/or the increase in the quantity of KT, both in the presence and absence of teeth or implants. In cases where an increase in KT is required, the gold standard is FGG [1-3]. This procedure always requires a second surgical site that serves as the KT donor area. This, of course, leads to an increase in morbidity and risk of complications [1,4,6]. To avoid tissue sampling (usually from the palate), numerous biological substitutes have been proposed (such as extracellular matrix membrane, bilayer collagen membrane, living cellular construct, and acellular dermal matrix). None of these, however, allow clinical results similar to the traditional technique and all, however, significantly increase the costs [4,5].

Platelet concentrates, widely used in medicine and dentistry, have also attracted the attention of researchers as a biological substitute in periodontology [6-10]. They allow high concentrations of autologous growth factors (GFs) to be delivered directly to host tissues during regenerative procedures. GFs act as chemotactic factors for various cell types (e.g., monocytes, fibroblasts, endothelial cells, stem cells, and fibroblasts) and stimulate the proliferation and differentiation of progenitor cells [11]. In particular, PRF is a method developed as an evolution of platelet-rich plasma, as a simplified preparation without biochemical manipulation of the blood [12]. In the literature, there is already much evidence that, in periodontal regeneration, PRF allows for a gain in KT, greater comfort for the patient, and a natural appearance (absence of patch-effect) [6,8,13-15] and resistance to infection [16].

Recently, new PRF preparation protocols have been proposed aimed at lengthening degradation times by heating the plasma obtained from horizontal centrifugation (H-PRF). The loss of GFs caused by heating is compensated by reincorporating the liquid PRF containing the cells extracted from the buffy coat into the heated plasma. The final product, therefore, would have great stability and solidity, preserving and releasing GFs for up to or beyond 10 days [17-20]. This would allow us to reduce the performance gap with FGG and therefore make the H-PRF technique a valid and simple alternative for VP and a new therapeutic tool in periodontology.

## Conclusions

The FGG is certainly a proven and effective technique for VP. The ever-increasing demand for comfort and rapid healing times, however, requires new approaches. The ability to transfer knowledge and techniques from one medical specialty to another allows you to always have new perspectives. The use of H-PRF allows the already notable results of periodontal surgery to be improved and made more comfortable. With all the limitations implicit in the presentation of a clinical case, the use of H-PRF in VP appears to be a valid option as an alternative to FGG. The result obtained appears acceptable as a KT gain, both in terms of quantity and aesthetics. Furthermore, the absence of a second surgical site certainly increases patient comfort and reduces risks and complications.

## References

[1] Kim DM, Neiva R (2015). Periodontal soft tissue non-root coverage procedures: a systematic review from the AAP regeneration workshop. J Periodontol.

[2] Ahmed S, Patel MAR, Haneef M (2020). Comparison between two surgical techniques for vestibuloplasty - a retrospective study. J Oral Med Oral Surg Oral Pathol Oral Radiol.

[3] Kungsadalpipob K, Supanimitkul K, Manopattanasoontorn S, Sophon N, Tangsathian T, Arunyanak SP (2020). The lack of keratinized mucosa is associated with poor peri-implant tissue health: a cross-sectional study. Int J Implant Dent.

[4] Dragan IF, Hotlzman LP, Karimbux NY, Morin RA, Bassir SH (2017). Clinical outcomes of comparing soft tissue alternatives to free gingival graft: a systematic review and meta-analysis. J Evid Based Dent Pract.

[5] Bertl K, Melchard M, Pandis N, Müller-Kern M, Stavropoulos A (2017). Soft tissue substitutes in non-root coverage procedures: a systematic review and meta-analysis. Clin Oral Investig.

[6] Temmerman A, Cleeren GJ, Castro AB, Teughels W, Quirynen M (2018). L-PRF for increasing the width of keratinized mucosa around implants: a split-mouth, randomized, controlled pilot clinical trial. J Periodontal Res.

[7] Tavelli L, McGuire MK, Zucchelli G, Rasperini G, Feinberg SE, Wang HL, Giannobile WV (2020). Biologics-based regenerative technologies for periodontal soft tissue engineering. J Periodontol.

[8] Mancini L, Tarallo F, Quinzi V, Fratini A, Mummolo S, Marchetti E (2021). Platelet-rich fibrin in single and multiple coronally advanced flap for type 1 recession: an updated systematic review and meta-analysis. Medicina (Kaunas).

[9] Tavelli L, Ravidà A, Barootchi S, Chambrone L, Giannobile WV (2021). Recombinant human platelet-derived growth factor: a systematic review of clinical findings in oral regenerative procedures. JDR Clin Trans Res.

[10] Salgado-Peralvo AO, Uribarri A, Kewalramani N, Peña-Cardelles JF, Liñares A (2023). The use of platelet-rich fibrin in vestibuloplasty: a 36-month follow-up technique report. Clin Adv Periodontics.

[11] Everts PA, Lana JF, Onishi K (2023). Angiogenesis and tissue repair depend on platelet dosing and bioformulation strategies following orthobiological platelet-rich plasma procedures: a narrative review. Biomedicines.

[12] Dohan DM, Choukroun J, Diss A, Dohan SL, Dohan AJ, Mouhyi J, Gogly B (2006). Platelet-rich fibrin (PRF): a second-generation platelet concentrate. Part I: technological concepts and evolution. Oral Surg Oral Med Oral Pathol Oral Radiol Endod.

[13] Castro AB, Meschi N, Temmerman A, Pinto N, Lambrechts P, Teughels W, Quirynen M (2017). Regenerative potential of leucocyte- and platelet-rich fibrin. Part A: intra-bony defects, furcation defects and periodontal plastic surgery. A systematic review and meta-analysis. J Clin Periodontol.

[14] Tarallo F, Mancini L, Pitzurra L, Bizzarro S, Tepedino M, Marchetti E (2020). Use of platelet-rich fibrin in the treatment of grade 2 furcation defects: systematic review and meta-analysis. J Clin Med.

[15] Chen L, Cheng J, Cai Y, Zhang J, Yin X, Luan Q (2023). Efficacy of concentrated growth factor (CGF) in the surgical treatment of oral diseases: a systematic review and meta-analysis. BMC Oral Health.

[16] Miron RJ, Fujioka-Kobayashi M, Bishara M, Zhang Y, Hernandez M, Choukroun J (2017). Platelet-rich fibrin and soft tissue wound healing: a systematic review. Tissue Eng Part B Rev.

[17] Fujioka-Kobayashi M, Schaller B, Mourão CFAB, Zhang Y, Sculean A, Miron RJ (2021). Biological characterization of an injectable platelet-rich fibrin mixture consisting of autologous albumin gel and liquid platelet-rich fibrin (Alb-PRF). Platelets.

[18] Gheno E, Mourão CFAB, Mello-Machado RC (2021). In vivo evaluation of the biocompatibility and biodegradation of a new denatured plasma membrane combined with liquid PRF (Alb-PRF). Platelets.

[19] Kargarpour Z, Nasirzade J, Panahipour L, Miron RJ, Gruber R (2020). Liquid platelet-rich fibrin and heat-coagulated albumin gel: bioassays for TGF-β activity. Materials (Basel).

[20] Zheng X, Yan X, Cheng K, Feng M, Wang Y, Xiao B (2022). Exploration of proper heating protocol for injectable horizontal platelet-rich fibrin gel. Int J Implant Dent.

